# Supplementary material for: Content-rich biological network constructed by mining PubMed abstracts
Source: BMC Bioinformatics. 2004 Oct 8;5:147. doi: 10.1186/1471-2105-5-147 (PMC528731; doi:10.1186/1471-2105-5-147)
Supplement: Additional File 2 — The original results of the above study (non-essential files are deleted to keep the file size under the limit set by BMC bioinformatics). [file 1471-2105-5-147-S2.bz2 › chilibotAdditionalFile2/dip05/516ID11390379E10819/html/FLNA_CASR.html]

 


 **FLNA** and **CASR** 
  
Found 2 abstracts in PubMed, retrieved 2.  
 

 What does Google say? 
 PDF only 
| .edu only 

---

**Interactive relationship** (e.g. stimulation, inhibition, etc)

**Neutral relationship**- Filamin A  [ **FLNA** ]  binds to the carboxyl terminal tail of the calcium sensing receptor  [ **CASR** ] , an interaction that participates in CaR mediated activation of mitogen activated protein kinase.  Ref: 11390380 J Biol Chem, 2001
